# Disease biomarker identification from gene network modules for metastasized breast cancer

**DOI:** 10.1038/s41598-017-00996-x

**Published:** 2017-04-21

**Authors:** Pooja Sharma, Dhruba K. Bhattacharyya, Jugal Kalita

**Affiliations:** 1 Tezpur University, Computer Science and Engineering Dept, Tezpur, Assam 784028 India; 2 Department of Computer Science, University of Colorado, Colorado Springs, United States

## Abstract

Advancement in science has tended to improve treatment of fatal diseases such as cancer. A major concern in the area is the spread of cancerous cells, technically refered to as metastasis into other organs beyond the primary organ. Treatment in such a stage of cancer is extremely difficult and usually palliative only. In this study, we focus on finding gene-gene network modules which are functionally similar in nature in the case of breast cancer. These modules extracted during the disease progression stages are analyzed using p-value and their associated pathways. We also explore interesting patterns associated with the causal genes, viz., SCGB1D2, MET, CYP1B1 and MMP9 in terms of expression similarity and pathway contexts. We analyze the genes involved in both the stages– non metastasis and metastatsis and change in their expression values, their associated pathways and roles as the disease progresses from one stage to another. We discover three additional pathways viz., *Glycerophospholipid metablism*, *h*-*Efp pathway* and *CARM1* and *Regulation of Estrogen Receptor*, which can be related to the metastasis phase of breast cancer. These new pathways can be further explored to identify their relevance during the progression of the disease.

## Introduction

A normal cell follows a well-known path of growth, divison and death although there are exceptions. Some cells do not die. They keep on dividing again and again, leading to abnormal growth. Such condition of cells is called cancerous, and it corresponds to a class of diseases known as cancer. Unrestricted growth of cells leads to the formation of lumps known as tumors, which can interfere in activities of bodily systems such as the nevous system or the circulatory system. These lumps of cells can be benign or malignant. Benign tumors do not disturb the normal functioning of the body. They are still usually controlled by genes controlling apoptosis. On the other hand, malignant tumor cells invade organs, thereby disrupting their normal functioning. They can also move throughout the body using the blood or the lymphatic system as the channel and invade other parts. Such spread of the disease is referred to as the metastatic stage. When cancerous cells metastasize, it becomes very difficult to treat them. The current characterization of this crucial stage of the disease is incomplete and as a result, proper diagnosis is often not feasible. Certain studies such as^1–3^ have analyzed differentially expressed genes for metastatic stages of lymphoma, lung cancer and leukaemia. Veer *et al*.^4^ and Wang *et al*.^5^ have identified 70 genes that may be associated with the metastatic stage of breast cancer. Chuang *et al*.^6^ use a protein-protein interaction network to determine the markers in the metastasis stage. All these studies focus on extracting individual disease markers responsible for disease progression. However, studies have shown that these genes may be associated with a variety of functions in the body, and interplay among the genes may lead to disorders that finally lead to cancer. Therefore, it would be beneficial to study the disease progression from the pathway point of view. This is because a biological pathway involves a number of molecules simultaneously, and these molecules have to work in coordination to perform normal cellular activities. A slight change in any of the molecules may lead others in the chain to behave abnormally, resulting in disease diagnosis.

Treating cancer for the non-metastatic stage is possible by drugs or by chemotherapy, but if the disease has spread to other organs, it becomes very difficult to diagnose on time and accordingly provide treatment. In this paper, we use the biological similarity between genes to identify functionally related modules. Gene expression data only consider the preparatory information available during the experiments. Analyzing the modules using only a part of the laboratory information would not be justifiable as genes are known to be functionally correlated in nature. Therefore, we use both topological and functional properties of genes to find biologically significant modules. Finding modules with high coherence is very essential in our case, as the main target of this work is to analyze the different characteristics of genes including the causal genes of breast cancer from different perspective. Such an analysis incorporating evidence from published literature can be used by biologists and other researchers to carry forward their work on these genes and their associated features.

This work provides a comprehensive study of biomarkers associated with breast cancer. In this paper, we make the following contributions.

- We propose an effective gene-gene network module extraction technique from micro-array gene expression data. We use both the toplogical and functional characteristics of the gene networks to find highly enriched modules.
- We analyze members of these modules from different perspectives, eg., expression patterns and pathways.
- We identify relevant pathways by analyzing modules for both the stages. Such pathways provide an insight into the role of each gene in the body and can be further extended to identify its role during disease progression.
- We discover certain interesting characteristics of causal genes such as MET and WARS.
- We find three more pathways to be associated with the metastasis stage of the disease. These pathways can be further explored to find their role in progression of the disease.

## Experimental Results

We implemented our network construction and module extraction method in MATLAB running on an HP Z 800 workstation with two 2.4 GHz Intel(R) Xeon(R) processors and 12 GB RAM, using the Windows7 operating system. The semantic similarity for a given pair of genes is found using the GOSemSim package^7^ in R.

### Parameter tuning for p-value computation

We carried out our experiments at various *CCT* and *SST* thresholds. *CCT* and *SST* are a clustering coefficient and a semantic similarity threshold respectively, set by the user. *CCT* takes care of the topological structure of the network and *SST* is concerned with the biological similarity among the nodes involved in the network. In order to choose the optimal value of the parameter, we compute the p-value for each module at different thresholds. This is because p-value gives the probability for a set of genes to be enriched with the same functional group. The *p*–*value* for a module *M* enriched with functional group *F* is given as

1 $$p-value=1-\sum _{i=0}^{q-1}\frac{(\begin{array}{c}|F|\\ i\end{array})(\begin{array}{c}|V|-|F|\\ |M|-i\end{array})}{(\begin{array}{c}|V|\\ |F|\end{array})}$$

where *M* contains *q* genes in *F* and entire network contains |*V*| genes.

To choose the best set of parameters, we compute the p-value of each module obtained at CCT values of 0.3, 0.5 and 0.7. The set of SST values is set to be 0.3, 0.5 and 0.7 for each run of CCT. The p–values of top three modules obtained at different threshold values are given Table 1.

**Table 1:** p–value of top 3 modules obtained using different thresholds in metastasis stage.

Modules	SST = 0.3 CCT = 0.3	SST = 0.5 CCT = 0.3	SST = 0.7 CCT = 0.3	SST = 0.3 CCT = 0.5	SST = 0.5 CCT = 0.5	SST = 0.7 CCT = 0.5	SST = 0.3 CCT = 0.7	SST = 0.5 CCT = 0.7	SST = 0.7 CCT = 0.7
M1	5.41E-6	2.25E-5	5.41E-6	4.14E-6	5.41E-6	5.10E-7	5.41E-6	2.25E-5	5.41E-6
M2	3.41E-5	3.41E-5	4.14E-6	5.41E-6	3.41E-5	7.14E-7	3.41E-5	1.56E-5	3.41E-5
M3	6.25E-5	1.68E-4	3.41E-5	3.41E-5	2.25E-5	8.41E-6	1.68E-4	1.68E-4	2.25E-5

In Table 1, we see that two of the three top modules show p-value of *5*.*10E*-*7* and *7*.*14E*-*7* at SST value of 0.7 with CCT value set to 0.5, while for the rest of the modules, it can be seen that the best p-value obtained is in the range of *E*-*6*.

Therefore, we can say that the optimal parameter set is achieved at CCT = 0.5 and SST = 0.7. This is supported by the fact that lower p-value signifies modules which are more biologically similar in nature among themselves and thus the ultimate purpose of module extraction is satisfied.

### Pathway identification from module members

We have carried out an extensive analysis of top five modules in terms of p-value in both non-metastasis and metastasis stages. We have identified the pathways to which each member gene belongs to in a given module. This is done using the DAVID tool^8^. The members of the modules along with the pathways they associate with are given in Table 2.

**Table 2:** Non-Metastasis and Metastasis modules along with pathway information and p-value.

Module No.: Members	*Non-metastasis modules*	Pathway associated gene names	p-value
	Pathways		
1: SRGN, MX1, GBP1, PLEK, PDE4B, SLA, IL32, CXCL9, RUNX3, IFI44L, CD52, LRMP, TRBV19, CTSS, PDE4B, CCL5, CXCL10, CCND2, CYP1B1, WARS, MMP9, PFKP, TAP1, ARHGAP4, SLC2A3	antigen processing and presentation	CTSS	5.38E-6
	purine metabolism	PDE4B	
	cytokine-cytokine receptor interaction, chemokine signalling pathway, NOD-like receptor signaling pathway, cytosolic DNA-sensing pathway, toll-like receptor signaling pathway.	CCL5	
	cytokine-cytokine receptor interaction, chemokine signalling pathway, NOD-like receptor signaling pathway, cytosolic DNA-sensing pathway, toll-like receptor signaling pathway, RIG-I-like receptor signaling pathway	CXCL10	
	p53 signaling pathway, Wnt signaling pathway, focal adhesion, Jak-STAT signaling pathway, cyclins and cell cycle regulation	CCND2	
	steroid hormone biosynthesis	CYP1B1	
	tryptophan metabolism, Aminoacyl-tRNA biosynthesis	WARS	
	leukocyte transendothelial migration, pathways in cancer	MMP9	
	glycolysis/gluconeogenesis pentose phosphate pathway, fructose and mannose metabolism, galactose metabolism	PFKP	
	antigen processing and presentation	TAP1	
	Rho cell motility signaling pathway	ARHGAP4	
	facilitated glucose transporter	SLC2A3	
2: CCL5, CCND2, WARS, LAG3	cytokine-cytokine receptor interaction, chemokine signalling pathway, NOD-like receptor signaling pathway, cytosolic DNA-sensing pathway, toll-like receptor signaling pathway.	CCL5	3.08E-5
	p53 signaling pathway, Wnt signaling pathway, focal adhesion, Jak-STAT signaling pathway, cyclins and cell cycle regulation	CCND2	
	tryptophan metabolism, aminoacyl-tRNA biosynthesis	WARS	
3: CCL5, CCND2, WARS, IGK@, IGLV5-45	cytokine-cytokine receptor interaction, chemokine signalling pathway, NOD-like receptor signaling pathway, cytosolic DNA-sensing pathway, toll-like receptor signaling pathway.	CCL5	1.68E-4
	p53 signaling pathway, Wnt signaling pathway, focal adhesion, Jak-STAT signaling pathway, cyclins and cell cycle regulation	CCND2	
	tryptophan metabolism, aminoacyl-tRNA biosynthesis	WARS	
4: CCL5, CCND2, WARS, IGLV3-19	cytokine-cytokine receptor interaction, chemokine signalling pathway, NOD-like receptor signaling pathway, cytosolic DNA-sensing pathway, toll-like receptor signaling pathway.	CCL5	1.68E-4
	p53 signaling pathway, Wnt signaling pathway, focal adhesion, Jak-STAT signaling pathway, cyclins and cell cycle regulation	CCND2	
	tryptophan metabolism, aminoacyl-tRNA biosynthesis	WARS	
5: CCL5, CCND2, WARS, NKG7	cytokine-cytokine receptor interaction, chemokine signalling pathway, NOD-like receptor signaling pathway, cytosolic DNA-sensing pathway, toll-like receptor signaling pathway.	CCL5	1.68E-4
	p53 signaling pathway, Wnt signaling pathway, focal adhesion, Jak-STAT signaling pathway, cyclins and cell cycle regulation	CCND2	
	tryptophan metabolism, aminoacyl-tRNA biosynthesis	WARS	
	***Metastasis modules***		
1: CCL5, CCND2, WARS, SRGN, TRAC,	cytokine-cytkine receptor interaction, chemokine signalling pathway, NOD-like receptor signaling pathway, cytosolic DNA-sensing pathway, toll-like receptor signaling pathway.	CCL5	5.10E-7
	p53 signaling pathway, Wnt signaling pathway, focal adhesion, Jak-STAT signaling pathway, cyclins and cell cycle regulation	CCND2	
	tryptophan metabolism, aminoacyl-tRNA biosynthesis	WARS	
2: WARS, PDYN, NUCB1, GUSBP3	tryptophan metabolism, aminoacyl-tRNA biosynthesis	WARS	7.14E-7
	opioid prodynorphin pathway, signaling by GPCR	PDYN	
3: WARS, ESR1, NUCB1, ASCL1	tryptophan metabolism, aminoacyl-tRNA biosynthesis	WARS	8.41E-6
	CARM1 and regulation of the Estrogen Receptor, h-Efp Pathway	ESR1	
4: CCL5, CCND2, WARS, SRGN, TRBV19	cytokine-cytkine receptor interaction, chemokine signalling pathway, NOD-like receptor signaling pathway, cytosolic DNA-sensing pathway, toll-like receptor signaling pathway.	CCL5	9.27E-6
	p53 signaling pathway, Wnt signaling pathway, Focal adhesion, Jak-STAT signaling pathway, cyclins and cell cycle regulation	CCND2	
	tryptophan metabolism, aminoacyl-tRNA biosynthesis	WARS	
5: CCL5, WARS, LCAT, MFGE8	cytokine-cytkine receptor interaction, chemokine signalling pathway, NOD-like receptor signaling pathway, cytosolic DNA-sensing pathway, toll-like receptor signaling pathway.	CCL5	1.52E-5
	tryptophan metabolism, aminoacyl-tRNA biosynthesis	WARS	
	glycerophospholipid metabolism	LCAT	

In Table 2, we see that three pathways viz., *Glycerophospholipid metablism*, *h*-*Efp pathway* and *CARM1 and Regulation of Estrogen Receptor* are associated with genes found in modules in the metastasis stage. These three new pathways can be further explored to find how they are responsible for spreading the disease to other organs.

**Figure 1:**
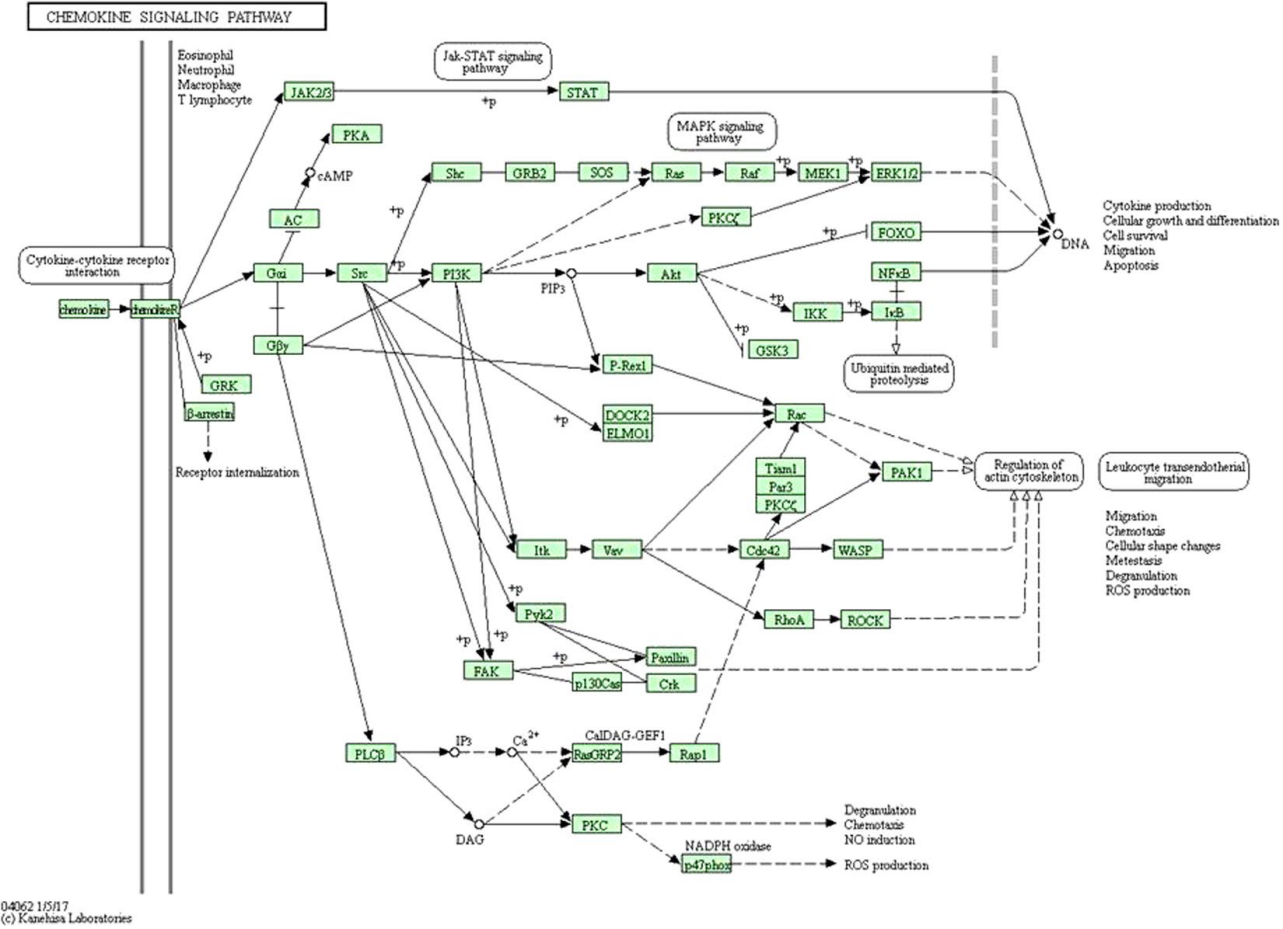
Chemokine signalling pathway (source-http://www.kegg.jp/kegg/kegg1.html).

**Figure 2:**
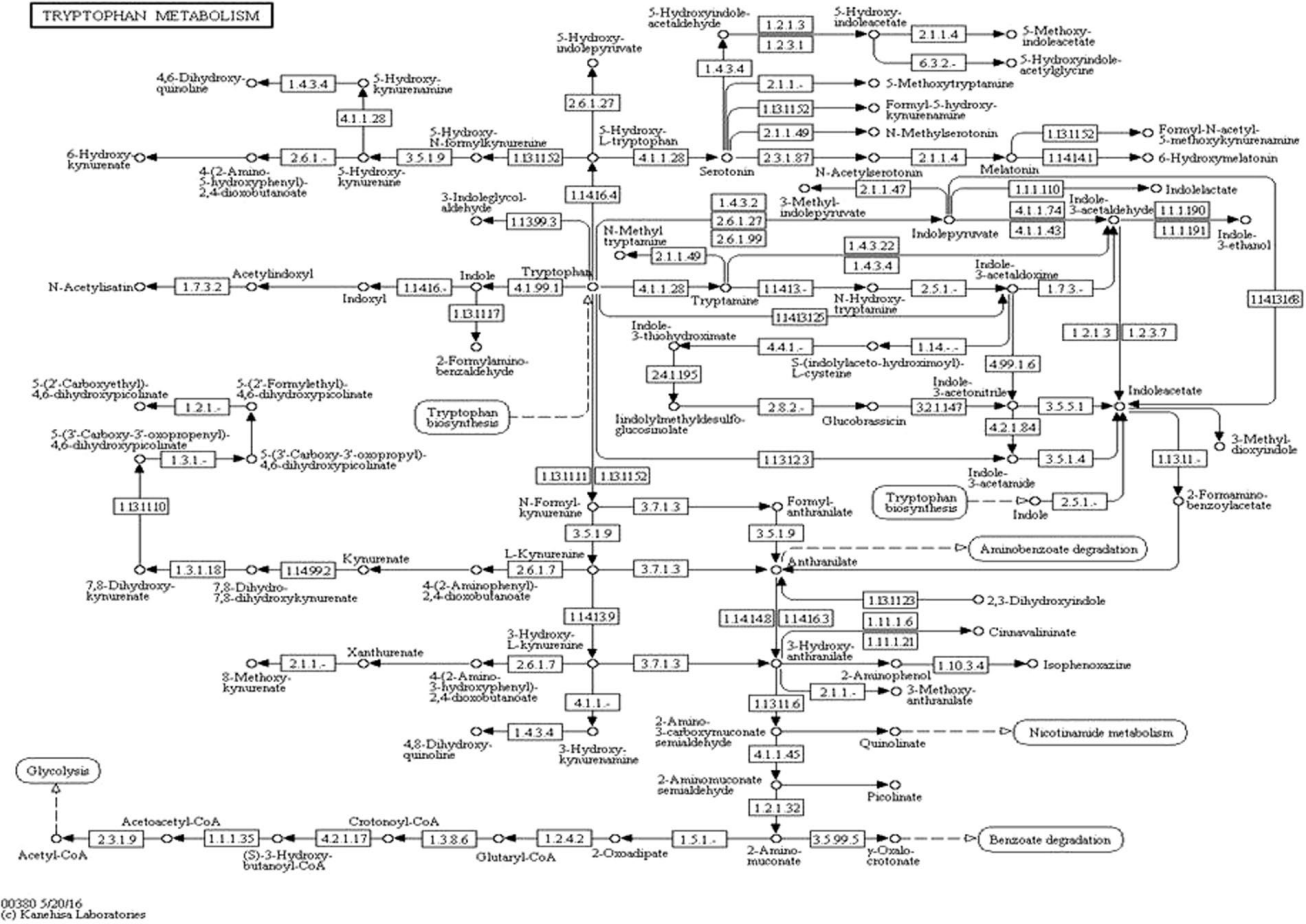
Tryptophan metabolism (source-http://www.kegg.jp/kegg/kegg1.html).

As explained in Proposition 2, we suggest that genes (other than the disease genes) found in a disease associated module also contribute to the disease. Let us consider the example of the top module in the metastasis stage. This module has CCL5, CCND2 (a disease gene), WARS, SRGN and TRAC as its member elements. In Table 2, we see the different pathways associated with its members. We provide pathways for only those genes which are found in literature. We see that the disease gene **CCND2** is related to *p53 signaling pathway*, *Wnt signalling pathway*, *focal adhesion*, *Jak*-*STAT signaling pathway* and others. Among its member genes, we find that **CCL5** is involved in *cytokine*-*cytokine receptor interaction*, *chemokine signalling pathway* etc. Looking into the pathway structure of *chemokine signaling pathway* given in the KEGG database^9^, we see that these two pathways are linked to the *Jak*-*STAT signalling pathway*, which is shown to be linked to the disease gene pathway. Figure 1 shows the *chemokine*-*signalling pathway*. The **WARS** gene belongs to the *trytophan metabolism pathway*, which aids in *glycolysis* as seen in Fig. 2. The *glycolysis* mechanism is indirectly regulated by the *Wnt signalling pathway* ^10^. This signalling pathway also corresponds to the disease gene pathway. Slight perturbation in any of these pathways may affect the changes to be carry forwarded, thus leading to certain disorders. Therefore, we can say that member genes of a disease module also contribute to the disease.

#### Interpreting utility of common genes in both stages

**Figure 3:**
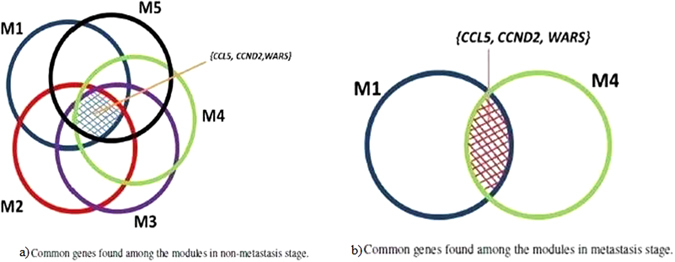
Common genes found among the modules in both the stages.

A Venn diagram representation of the common genes found among the modules in both the stages is shown in Fig. 3. We see that genes CCL5, CCND2 and WARS are found among all the five modules in non-metastasis stage. However, in metastasis stage, Fig. 3(b), we see that these three genes occur simultaneously in modules M1 and M4 only. Among the rest of the modules, only the WARS gene is present as a common element. The other two genes are not associated with three of the modules in the metastasis stage. This may be due to its low semantic value with the seed node for the respective modules. In addition to the disease gene, two other genes are found to be strongly related to the breast cancer disease. CCND2, which is the causal gene found in both stages is known to have a higher invasive ability. Certain results also suggest that overexpression of this gene in carcinomic cells has an enhanced effect on *in vivo* aggressive growth pattern^11^. CCL5/CCR3 signalling actively encourages metastasis by polarization of CD4+T cells, for luminal breast cancer^12^. WARS, popularly known as Tryptophanyl-tRNA synthetase corresponds to the aminoacyl-tRNA synthetase family. They are involved in RNA transcription, protein synthesis and in angiogenic signalling pathways^13^. Overexpression of tRNA synthetase promotes migratory movements of carcinogenic cells^14^. This may be one of the reasons why WARS is present in all modules of both the stages.

### Common pathways among modules and change in expression value of associated genes

We also analyze the common pathways associated with the common genes in both the stages and observe the changes in their expression values as the disease progresses from the non-metastatic to the metastatic stage. This is shown in Table 3. Among all the genes which are common to the two stages, they show a decrease in their expression value in the two stages.

**Table 3:** Expression value of genes involved in common pathways in both the stages.

Pathway	Commongene(s)	Non-Metastasis		Metastasis		Change
		Module No.	Expression Value	Module No.	Expression Value	
Cytokine-cytokine pathway	CCL5	14, 6, 15, 21	9.29	12, 15, 10	8.826	↓
Chemokine signaling pathway	CCL5	14, 6, 15, 21	9.29	12, 15, 10	8.26	↓
p53signaling pathway	CCND2	47, 14, 6, 15, 21	5.961	12, 15	5.676	↓
Cytosolic DNA sensing pathway	CCL5	14, 6, 15, 21	9.29	12, 15, 10	8.26	↓
Wnt signaling pathway	CCND2	47, 14, 6, 15, 21	5.961	12, 15	5.676	↓
Tryptophan metabolism	WARS	14, 6, 15, 21	8.65	12, 13, 8, 15, 10	8.276	↓
Focal adhesion	CCND2	47, 14, 6, 15, 21	5.961	12, 15	5.676	↓
Toll-like receptor signaling pathway	CCL5	14, 6, 15, 21	9.29	12, 15, 10	8.26	↓

The role of these common pathways in terms of the disease is highlighted here.

- *Cytokine*-*cytokine pathway*: Cytokines are released whenever there is some infection or inflammation so as to hamper the tumor’s development. Carcinogens can also respond to the host with cytokines so as to promote growth and spreading of the disease to other organs^15^.
- *Chemokine signaling pathway*: Expression values of chemokines are changed in many forms of malignancies and eventually lead to disturbed chemokine signalling pathway^16^.
- *p53signaling pathway*: p53 loss can disrupt metastasis related pathways. However, transcriptionally defective TP53 mutants can promote metastasis stage of cancer^17^.
- *Wnt signaling pathway*: Activity of the Wnt/*β*-catenin signalling pathway plays a significant role during the development of breast cancer^18^.
- *Tryptophan metabolism*: Higher expression of enzymes involved in tryptophan degradation is known to be associated with several forms of cancer such as lung cancer, breast cancer and melanoma^19^.
- *Toll*-*like receptor signaling pathway*: Toll like receptor signaling in tumor cells play a role in aggressive behavior of tumor by excessive secretion of cytokines/chemokines^20^.
- *Cytosolic DNA sensing pathway*: The role of this pathway is yet not clear in context of the disease, but since it is found in both the stages, we can say that it plays a significant role in causing the disease.

### Non-participation of some causal genes in the modules of metastasis

Analyzing the characteristics of the top five modules obtained in both the stages, we find that CCND2 is found in most of the modules. This gene has been reported to be a causal gene by GeneCard^21^. A few other disease genes such as XBP1, SCGB1D2, MET, CYP1B1 and MMP9 have been reported to be active during the non-metastasis module formation. However, these genes do not participate in the metastasis stage of module formation, although they seem to be actively involved during the non-metastasis module formation process. Based on our experimental observations as well as analysis of the literature, we put forward Proposition 1.

**Proposition 1**. *A causal gene* ${g}_{i}\in {m}_{i}^{nm}$, *the i* ^*th*^ *non-metastasis module may not participate in coherent module formation at metastatic stage*.

**Explanation:** Assume that a gene ${g}_{i}\in {m}_{i}^{nm}$ also $\in {m}_{j}^{m}$, the *j* ^*th*^ module in metastatic stage. It is evident from the literature and also from our experimental study that during stage transistion from non-metastasis to metastasis, often a gene undergoes significant variation (i.e., fall or rise) in expression or semantic similarity values. Such a gene with signficantly varied expression or semantic similarity values may not remain coherent with other module forming genes, and hence may not satisfy the *CCT* and *SST* cutoffs, and may not participate in module formation during the metastasis stage. It contradicts our assumption and hence the proof.

We analyzed the semantic similarity values among these five genes and found that XBP1, MET and CYP1B1 are probable candidates for module formation. However, these could not be members of the modules in the metastasis stage probably due to their low semantic similarity score with the seed nodes.

### Analyzing genes based on expression values

**Figure 4:**
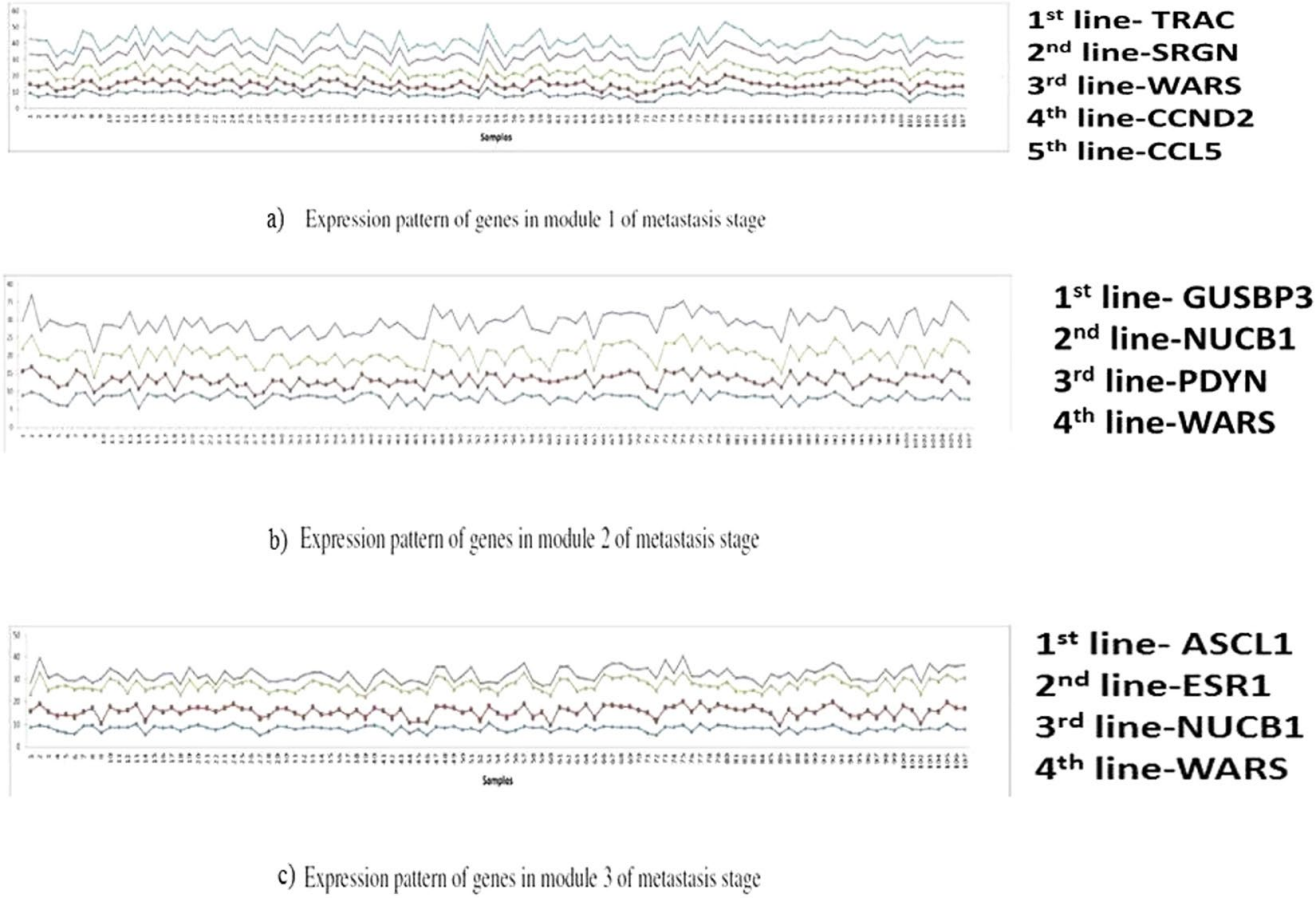
Expression patterns of genes in top three modules of metastasis stage.

**Figure 5:**
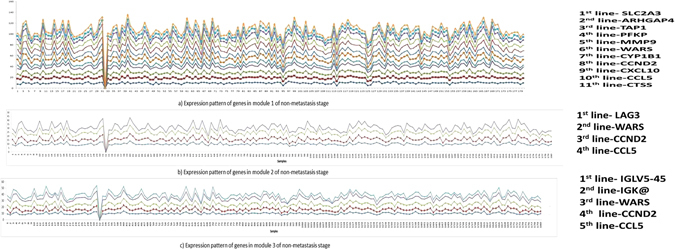
Expression patterns of genes in top three modules of non-metastasis stage.

Apart from analyzing expression pattern of only the common genes found among the modules across the stages, we also tried to find if genes involved during module formation are coherent among themselves or not. We show the expression patterns of genes in the top three modules obtained during both the stages in Figs 4 and 5. In these figures, we see that participating genes demonstrate high coherence across the samples.

**Figure 6:**
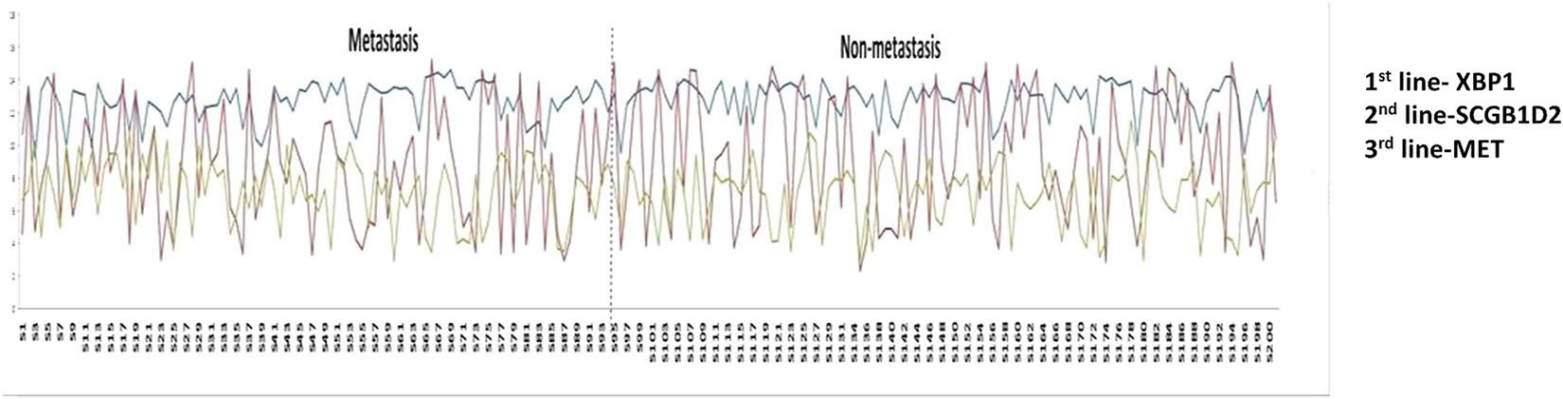
Expression pattern of causal genes in both stages.

We then observe expression patterns of genes associated with the disease as given in GeneCard. These genes are referred to as causal genes. The average expression levels of these genes across the two stages are analyzed and we find that CYP1B1 and MMP9 show an *increasing* trend in their average expression values from non-metastasis to metastasis stage. This is clearly different from the normal trend of expression values during progression of diseases. The other three genes XBP1, SCGB1D2 and MET show a *decreasing* trend during the progression phase. We plot the expression patterns of these three genes across the two stages in Fig. 6. Two of the genes SCGB1D2 and MET show normal trend as seen among the rest of the genes. However, XBP1 shows a peculiar trend in both stages. The expression values of this gene shows very low variations across all the 286 samples. This may be due to its inherent nature, as it acts as a transcription factor which regulates the gene expression levels for the immune system and in other cellular responses. This may be one of the reasons of its low variation during disease progression as the immune system prompts the body to respond equally during the non-metastasis and metastasis stages.

## Discussion

Cancer is a complex disease. Its distinguishing characteristics can best be represented in the form of a modular structure, technically referred to as a cancer hallmark network. Earlier studies have revealed six types of hallmarks associated with cancer^22^. These are (i) cancerous cells that promote their own growth, (ii) they thwart inhibitory signals that would harm them, (iii) they do not take part in  from programmed cell death, (iv) they promote growth and functioning of blood vessels which provide nutrition to the cancerous cells, (v) they have a tendency to keep on multiplying and (vi) they move out to other organs thereby spreading the disease. Four new characteristic features have been discovered to be associated with cancerous cells^23^. These were added to the hallmarks of cancer: (vii) associated pathways show abnormality, (viii) cancer cells remain hidden from the immune system surveillance, (ix) abnormalities occur in the number of chromosomes in the cancerous cells and (x) there is inflammation in the affected tissue. For a cancerous cell to survive, divide and move out, it needs to have functional capabilities acquired through activation of different hallmarks during different times of cancerous progression^24, 25^.

An extensive study of the signalling network along with the tumor-genome sequencing output has illustrated the importance of metamorphing in case of tumor suppressor genes and their role in cancer development and progression over stages^26^. Mutation in these genes results in genome instability and chromosome amplification. Such a situation gives rise to heterogeneity in the tumor cells or the cancerous cells. This heterogeneity leads to loss of robustness among the disease biomarkers. It poses a major hurdle in cancer biomarker discovery as the contemporary methods of discovering biomarkers based on gene expression profiles are inadequate^27^. Identification of robust cancer biomarkers has recently gained importance in the research community. Methods such as MSS (Multiple Survival Screening)^26^ and ensembling methods^28^ work by characterizing cancer samples into various classes, depending on their gene expression profiles or signalling network structure. Once they are narrowed down to sub-classes, one can easily predict the associated biomarkers depending on their class characteristics.

The module extraction method proposed here is based on semantic similarity among the genes. This method extracts biologically similar modules as suggested by their p-values. Our module extraction method is used over the metastatic and non-metastatic stages of the breast cancer gene expression dataset. We have analyzed members obtained from the top five modules in terms of expression similarity and pathway point of view.

Certain interesting behaviors of the causal genes which are members such as *SCGB1D2*, *MET*, *CYP1B1* and *MMP9* are also studied. We have also suggested reasons for their peculiar behavior.

We have also identified reasons why certain genes are found in modules of only one stage. Apart from this, we give justification for how genes in a module coordinate with others to achieve the function associated with the module. We also justify why certain genes such as **WARS** are present in all identified modules in both the stages.

In addition to this, we analyze the expression values of the common genes in both stages of the disease and also speculate how these genes play role in causing the disease considering the pathway.

Lastly, we also find three new pathways namely *Glycerophospholipid metablism*, *h*-*Efp pathway* and *CARM1 and Regulation of Estrogen Receptor* to be associated with the metastasis stage of the disease. Understanding these pathways will give better insight into the progression of the disease.

## Materials and Method

Besides skin cancer, breast cancer is the most commonly dignosed disease among women in the United States. About 40,610 women in the U.S. are expected to die in 2017 from breast cancer^29^. If the disease is detected while still at the *in*-*situ* stage, the chance of survival is close to 100%. However, if the disease is left untreated, it usually spreads to other parts of the body (metastasize), and the first place it usually spreads is to the lymph nodes in the axilla area^30^. Metastatic breast cancer cells differ from the primary ones in properties such as receptor status. The cells often develop resistance to several lines of previous treatment and acquire special properties that permit them to metastasize to distant sites. Metastatic breast cancer can be treated, sometimes for many years, but it cannot be cured. Women in this stage of the disease have less than 16% chances of survival^31^. Therefore, it is very crucial to study the progression of the disease to this stage. We make one such effort here, and the findings of our work can be used by the biologists to better interpret the causes of the disease, to aid on-time treatment.

### Dataset Used

The study of progression of metastasis cancer from the non-metastasis stage requires a thorough understanding of the genes which get mutated during the disease progression, how their expression values change from time to time, what other changes are brought in during the progression, etc. In order to get a vivid picture of the disease progression, we perform our experiments on the GSE 20304 dataset on breast cancer relapse free suvival, obtained from ref. 32. It comprises of expression values of 286 patients. Among the 286 samples, 106 samples are from the metastatic stage and the rest are from the non-metastatic stage.

### Preprocessing and Sample selection

The dataset consists of 22,273 genes with expression values. In order to scale the values obtained over the large number of samples, we use *log2* transformation. We then use variance measure to decide upon the genes which are actively involved across all the 286 samples. We tune variance threshold *Vth* in the range 0.9–1.5, and accordingly we obtain seven different samples of genes, each with 7900, 7000, 6129, 5292, 4529, 3903 and 3356 genes respectively as given in Table 4.

**Table 4:** Various gene samples drawn at different *Vth* threshold.

	*Vth* = 0.9	*Vth* = 1.0	*Vth* = 1.1	*Vth* = 1.2	*Vth* = 1.3	*Vth* = 1.4	*Vth* = 1.5
Number of genes	7900	7000	6129	5292	4529	3903	3356

From our observation, we find the best suitable range to be 1.1–1.3. We carry out our module formation process using genes at this range. Modules obtained in this range are analyzed, and we find slightly higher number of modules at *Vth* = 1.1 than at *Vth* = 1.2 or *Vth* = 1.3. This may be a result of the extra 872 genes at this sample stage, which are later removed in the other two stages due to its low variance as compared to *Vth*. For our extensive analysis purpose, we use module results obtained at *Vth* = 1.2, as it is the mid-point of the two values. Apart from being the average of the two values, it also shows higher number of common modules when compared with the modules obtained at both the ends. This threshold value is also supported by Wang *et al*. in their work^33^, where they use it to get the maximum variance of genes among the samples for the dataset.

### Network construction and module extraction

The process of network construction begins with 5292 genes. Two different networks are constructed, one for non-metastatic stage and the other for the metastatic stage. These two networks are obtained based on the patient’s characteristics. The network A(i, j) is constructed using the Pearson’s correlation coefficient between the genes, PrC(i, j), where

2 $${A}_{(i,j)}=(\begin{array}{ll}1 & if\,{\rm{PrC}}(i,j)\ge 0.5\\ 0 & {\rm{otherwise}}.\end{array}$$

We choose 0.5 as the threshold for Pearson coefficient because we want to extract subnetworks or modules with balanced contributions from both gene expression and semantic similarity. Once the networks, i.e., *A*_*nm*_ for non-metastatic stage and *A*_*m*_ for metastatic stage are ready, we start with a seed growing technique to find modules. Seed selection for modules is based on the clustering coefficient of each gene in the network. The choice of seed is based on the node with the maximum clustering coefficient, such that this is greater than an input threshold value for CCT. In order to grow the seed, we use a second criterion which is the semantic similarity between gene pairs so as to find their relation with each other. We choose semantic similarity between gene pairs in order to get functionally relevant modules. The more biologically relevant a module is, the more will be its correspondence to the disease. This statement is supported by Proposition 2.

**Proposition 2**. *If for a given disease D*_*i*_, *the causal gene* ${g}_{i}\in {m}_{i}$, *i*.*e*., *i* ^*th*^ *network module of high biological significance*, *then any other gene* ${g}_{j}\in {m}_{i}$ *will also have a correspondence with D*_*i*_.

**Explanation:** A pair of genes (*g*_*i*_, *g*_*j*_) will be members of a module, *m*_*i*_ of high biological significance (i.e., with very low p-value) iff *PrC*(*g*_*i*_, *g*_*j*_) ≥ 0.5 and *SS*(*g*_*i*_, *g*_*j*_) ≥ *SST*. So, any other member gene of *m*_*i*_ will share highly similar functionality with gene *g*_*i*_ or *g*_*j*_. Therefore, since *g*_*i*_ corresponds to a disease *D*_*i*_, other member genes, say *g*_*j*_ ∈ *m*_*i*_ will also correspond to *D*_*i*_^34–36^, and hence the proof.

This proposition can be better understood with the help of an example given in Section *Pathway identification from module members*. In order to get the best semantic similarity between a pair of genes, we use Wang’s semantic similarity, which is known to be the best among all the existing measures^37, 38^. However, we have restricted ourselves to only those genes during expansion whose semantic similarity value is atleast equal to SST, set by the user. The parameter is set experimentally as discussed in the Section *Parameter tuning for p*-*value computation*. We now define some terms which are used during the module extraction process.

**Definition 1** (Semantically connected). *Two genes g*_*i*_ *and g*_*j*_ *are said to be semantically connected iff PrC*(*g*_*i*_, *g*_*j*_) ≥ *CCT and SS*(*g*_*i*_, *g*_*j*_) ≥ *SST*, *where CCT ad SST are user-defined thresholds*.

**Definition 2** (Clustering coefficient). *For an undirected graph*, *clustering coefficient of a node v*_*i*_ (*representing a gene*, *say g*_*i*_) *is the ratio of the number of links the node has among its neighbors*, *N*_*l*_ *to the total number of possible links for that node*. *Mathematically*,

3 $$CC({v}_{i})=\frac{2\times {N}_{l}}{{d}_{{v}_{i}}\times ({d}_{{v}_{i}}-1)}$$

*where* ${d}_{{v}_{i}}$ *is the degree of node v*_*i*_.

**Definition 3** (Seed node). *A node v*_*i*_ *is said to be a seed node if* $\forall {v}_{j}$, $CC({v}_{i}) > CC({v}_{j})$, *where* ${v}_{j}\in \{V-{v}_{i}\}$ *snd V is the set of nodes*.

**Definition 4** (Module). *A module is a set of nodes*/*genes which are semantically connected among each other*.

The steps for module extraction is given in Algorithm 1.

**Lemma 1**. *Two genes* (*g*_*i*_, *g*_*j*_) *are functionally coherent if they have high expression and semantic similarity*.

*Proof*. This proof is trivial because a pair of genes sharing high expression and semantic similarity show similar trend and are more functionally coherent. ☐

**Lemma 2**. *Nodes included by our method in a module have high functional coherence*.

*Proof*. A gene *g*_*i*_ can be a member of module, *m*_*i*_ iff *Expression*(*g*_*i*_, *g*_*j*_) ≥ 0.5 and *SS*(*g*_*i*_, *g*_*j*_) ≥ *SST*, where SST is the user-defined threshold for Semantic similarity. So, for any two member genes of a module, the expression similarity and semantic similarity between them is always high. Hence, the nodes in a module have high functional coherence.☐

**Table:** 

**Algorithm 1:** Algorithm for module extraction from gene gene network
**Input**: *A* = {*V*, *E*} (Gene gene network); *CCT* (Clustering coefficient threshold); *SST* (Semantic similarity threshold); *NSS* (Semantic similarity score matrix)
**Output**: *Modules* = {*C*_1_, *C*_2_, …, *C*_*N*_}, (a set of *N* modules)
1 Initialize clusterExpNode = *V*, Modules = *NULL*;
2 **while** |clusterExpNode| > 4 **do**
3 choose ${v}_{m}\in clusterExpNode$ such that $\forall {v}_{n}\in clusterExpNode$, *CC*(*v*_*m*_) ≥ *CC*(*v*_*n*_) and *CC*(*v*_*m*_) ≥ *CCT*; $partialCluster=partialCluster\cup {v}_{m}$;
4 **while** *v*_*m*_ *exists* **do**
5 choose another *v*_*i*_ from ${N}_{s({v}_{m})}$ if and only if $\exists {v}_{x}\in partialCluster$ such that *NSS*(*v*_*i*_, *v*_*x*_) ≥ *SST*
6 $$partialCluster=partialCluster\cup {v}_{i}$$;
7 *clusterExpNode* = *clusterExpNode* − *v*_*i*_;
8 $${N}_{s({v}_{m})}=({N}_{s({v}_{m})}\cup {N}_{s({v}_{i})})$$ choose next *v*_*i*_;
9 **end**
10 Mark *partialCluster* as *C*_*count*_ only when |*partialCluster*| ≥ 3;
11 $$Modules=Modules\cup {C}_{count}$$;
12 *count* + +;
13 **end**
14 Return *Modules*;

### Complexity Analysis

The module extraction technique involves selection of seed nodes based on the clustering coefficient of the nodes. This involves identifying neighbors of each node, which takes *O*(*n*) time in the worst case. To identify the number of links present among the neighbor sets takes another *O*(*n*) time. Thus, the seed selection process takes $O(n)\times O(n)\equiv O({n}^{2})$ time. The module expansion process starts from the node with the highest clustering coefficient. Sorting the nodes in terms of decreasing clustering coefficient requires *O*(*nlogn*) time and then comparing the top most node with user defined *CCT* requires *O*(1) time. Expansion of modules requires *O*(*n* ^2^) as it requires computing the neighbor set again for the seed node, which takes *O*(*n*) time and then finding its corresponding semantic similarity, which takes at most *O*(*n*) time. This semantic similarity value has to be compared with the *SST* value defined by the user, which takes *O*(1) time. Therefore, the overall time complexity for module extraction is $O({n}^{2})+O(nlogn)+O\mathrm{(1)}+O({n}^{2})+O(1)\equiv O({n}^{2})$.
